# Identification of biomarkers of chronic kidney disease among kidney-derived proteins

**DOI:** 10.1186/s12014-021-09340-y

**Published:** 2022-01-11

**Authors:** Kazuma Higashisaka, Sonoko Takeya, Haruhiko Kamada, Masanori Obana, Makiko Maeda, Mai Kabayama, Koichi Yamamoto, Nanan Ishida, Ryo Isaka, Hirofumi Tsujino, Kazuya Nagano, Noriyuki Tomiyama, Hiromi Rakugi, Yasushi Fujio, Kei Kamide, Yasuo Tsutsumi

**Affiliations:** 1 Laboratory of Toxicology and Safety Science, Graduate School of Pharmaceutical Sciences, Osaka University, 1-6, Yamadaoka, Suita, Osaka 565-0871 Japan; 2 Laboratory of Biopharmaceutical Research, National Institutes of Biomedical Innovation, Health and Nutrition, Ibaraki, Osaka Japan; 3 Center for Drug Design Research, National Institutes of Biomedical Innovation, Health and Nutrition, Ibaraki, Osaka Japan; 4 Global Center for Medical Engineering and Informatics, Osaka University, Suita, Osaka Japan; 5 Laboratory of Clinical Science and Biomedicine, Graduate School of Pharmaceutical Sciences, Osaka University, Suita, Osaka Japan; 6 Advanced Research of Medical and Pharmaceutical Sciences, Graduate School of Pharmaceutical Sciences, Osaka University, Suita, Osaka Japan; 7 Department of Health Promotion Sciences, Division of Health Sciences, Osaka University Graduate School of Medicine, Suita, Osaka Japan; 8 Department of Geriatric and General Medicine, Osaka University Graduate School of Medicine, Suita, Osaka Japan; 9 Department of Radiology, Osaka University Graduate School of Medicine, Suita, Osaka Japan

**Keywords:** Biomarker, Chronic kidney disease, Kidney-derived proteins

## Abstract

**Background:**

Chronic kidney disease (CKD) has few objective symptoms, and it is difficult to make an early diagnosis by using existing methods. Therefore, new biomarkers enabling diagnosis of renal dysfunction at an early stage need to be developed. Here, we searched for new biomarkers of CKD by focusing on kidney-derived proteins that could sensitively reflect that organ’s disease state.

**Methods:**

To identify candidate marker proteins, we performed a proteomics analysis on renal influx and efflux blood collected from the same individual.

**Results:**

Proteomics analysis revealed 662 proteins in influx blood and 809 in efflux. From these identified proteins, we selected complement C1q as a candidate; the plasma C1q level was significantly elevated in the renal efflux of donors. Moreover, the plasma concentration of C1q in a mouse model of diabetic nephropathy was significantly increased, in association with increases in blood glucose concentration and urinary protein content. Importantly, we demonstrated that the tendency of C1q to increase in the plasma of CKD patients was correlated with a decrease in their estimated glomerular filtration rate.

**Conclusion:**

Overall, our results indicate that our approach of focusing on kidney-derived proteins is useful for identifying new CKD biomarkers and that C1q has potential as a biomarker of renal function.

**Supplementary Information:**

The online version contains supplementary material available at 10.1186/s12014-021-09340-y.

## Background

Chronic kidney disease (CKD) is recognized as a leading public health problem worldwide, with a global estimated prevalence of 13.4% [1, 2]. The loss of kidney function in CKD is progressive and irreversible [3]. Renal replacement therapies, including dialysis and kidney transplantation, are necessary for patients who progress into end-stage renal disease [4]. Because the therapeutic goal of CKD is to avoid the need for renal replacement therapy, early diagnosis of renal dysfunction and prompt elimination of the causes of dysfunction are crucial.

However, the number of CKD patients is steadily increasing year by year, and it will continue to increase [5]. This is because CKD has few symptoms, and early diagnosis is difficult with existing methods [6]. In general, renal function is evaluated by using the glomerular filtration rate, which is the amount of plasma filtered from all glomeruli of the kidney per unit time. Alternatively, physical changes in urinary levels of protein and albumin, which are test items in urinalysis, are detected daily; there are therefore many false positives, and it is difficult to specifically diagnose CKD. In addition, when the blood creatinine level exceeds the reference hematology value, renal function has already decreased to about 40%, and it is difficult to detect the decrease in renal function at the initial stage [7]. Early identification of CKD is an important unmet medical need, not only for predicting and preventing CKD progression but also for improving patient survival and decreasing associated morbidities.

For the purpose of diagnosis of CKD, many attempts have been made to search for biomarkers by comparing variable expression levels of proteins in serum or urine between healthy subjects and patients [8, 9]. However, it is difficult to narrow down the protein or proteins specific to the disease because it cannot be determined whether protein level variations are caused by renal dysfunction or simply by individual differences such as genetic factors and environmental background. From this viewpoint, we focused on kidney-derived proteins, which are considered to be able to sensitively reflect the state of the kidneys [10], to search for CKD biomarkers that accurately reflect renal function. In addition, we thought that it would be possible to eliminate interindividual differences, which have been a problem in proteome analysis, by comparing renal influx and efflux blood samples collected from the same individual to identify kidney-derived proteins.

Here, we attempted to identify CKD biomarkers by focusing on kidney-derived proteins and validating the expression changes of the identified proteins in an animal model with renal dysfunction.

## Methods

### Collection of renal influx and efflux blood samples

Blood samples were collected at the time of adrenal vein sampling, which is used to definitively diagnose primary aldosteronism. From each of seven participants, renal efflux blood was collected from the left renal vein, and renal influx blood was collected from the femoral artery, which we assumed had the same composition as the blood entering the kidney. Each sample was collected by using a blood collection tube containing a serum separator; the obtained serum was stored at − 80 °C until analysis.

### Proteomics analysis

Each serum sample was centrifuged in 15,000 g for 5 min at 4 °C, applied to a 0.45-μm spin filter, and centrifuged in same condition again. To remove nonrenal-specific proteins present in large amounts in the serum, negative selection was performed via high-performance liquid chromatography (HPLC) by using a MARS Column Human-14 (Agilent Technologies, Santa Clara, CA, USA). To each 50-μg sample, we then added 0.25 M dithiothreitol followed by 0.375 M iodoacetamide. We incubated the mixture for 20 min in the dark at room temperature, added 0.2 μg trypsin, and incubated the mixture at 37 °C overnight. After the addition of 1% trifluoroacetic acid to each sample, digested peptides were desalted, purified, and concentrated with C18 microcolumns (Varian, Palo Alto, CA, USA). After lyophilization, the peptides were separated by HPLC with ion-exchange column. The supernatant underwent mass spectrometry (MS) by using a Q Exactive mass spectrometer (Thermo Fisher Scientific, Waltham, MA, USA). Identified peptides were accepted at a false discovery rate of < 1%, and protein identification through a Mascot search of the Swiss-Prot database accounted for oxidation of methionine residues and thiomethylation of cysteine residues via iodoacetamide.

### Assessment of association of C1q with CKD in mice

For the unilateral ureteral obstruction (UUO) model, C57BL/6 J male mice (n = 4–5, age, 8 weeks) were anesthetized under isoflurane and the left ureter was accessed dorsally and ligated (or not, for sham-operated controls) [11]; blood was collected from the abdominal aorta 7 days after ligation. For streptozocin (STZ)-induced diabetic mice, C57BL/6 J male mice (n = 5–6, age, 8 weeks) received STZ (50 mg/kg, Sigma-Aldrich, St. Louis, MO, USA) or citrate buffer (pH 4.5) intraperitoneally once daily for 5 consecutive days; 11 weeks after the last dose of STZ, blood was collected from the abdominal femoral artery. The urinary protein level in each mouse was measured by using Micro TP-Test photometric (FujiFilm Wako Pure Chemical Corporation, Osaka, Japan) according to the manufacturer’s protocol. Blood glucose concentration was measured by using a LabAssay Glucose kit (FujiFilm Wako Pure Chemical Corporation) according to manufacturer’s protocol.

### Collection of human peripheral blood samples

Serum from blood samples that had been collected from random patients (Table 1) was stored at − 80 °C until analysis. None of three non-CKD patients had proteinuria, and the estimated GFR exceeded 60 ml/min in all 3 of these patients. In addition, two of these three patients had diabetes mellitus.

**Table 1:** Clinical characteristics of non-CKD patients and patients with CKD

	Patient no.							
	1	2	3	4	5	6	7	8
Age	48	76	71	88	84	67	85	68
Sex	M	M	F	M	M	M	F	M
Diabetes mellitus	No	Yes	Yes	Yes	Yes	Yes	Yes	No
CKD	No	Yes	No	Yes	No	Yes	Yes	Yes
eGFR (mL/min/1.73 m^2^)	65	58.5	75.1	40.3	76.7	81	65.3	26.9
Proteinuria	No	Yes	No	No	No	Yes	Yes	Yes

### Quantitative analysis of C1q

Serum levels of C1q were measured by using commercial enzyme-linked immunosorbent assay (ELISA) kits according to the manufacturer’s instructions. For measuring serum levels of C1q in human, the ELISA kit was purchased from Abcam (Cambridge, United Kingdom); for mouse serum, the ELISA kit was purchased from R&D Systems (Minneapolis, MN, USA).

### Statistical analyses

Statistical analyses were performed by using Ekuseru–Toukei 2008 software (Social Survey Research Information, Tokyo, Japan). All data are expressed as means ± S.E.M. and were compared by using *t*-tests; *P* < 0.05 was considered significant.

## Results

### Identification of kidney-derived proteins

**Fig. 1:**
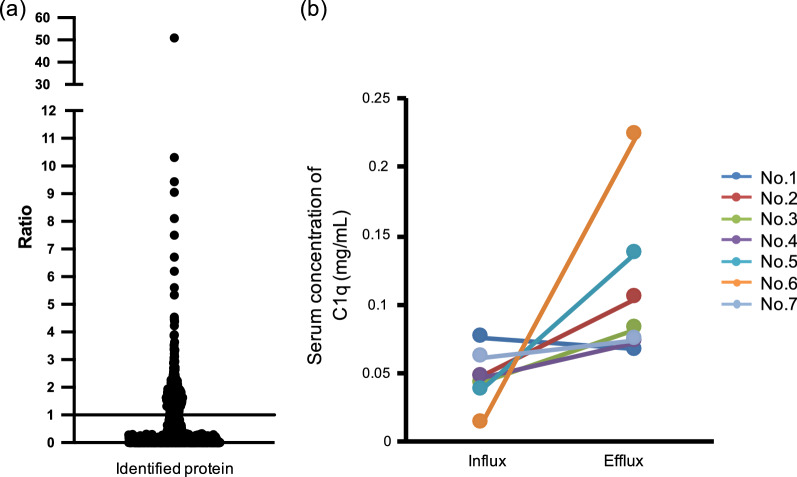
Concentrations of C1q in renal influx and efflux blood samples from human individuals. **a** Averaged ratio of each protein’s expression level in the efflux sample to its expression in the influx from all seven individuals. **b** C1q concentrations in renal influx and efflux blood samples from each of seven individuals (nos. 1–7) were examined by enzyme-linked immunosorbent assay (ELISA)

To identify kidney-derived proteins, renal influx and efflux blood samples were collected from seven human individuals and underwent proteomic analysis by LC–MS/MS. Comprehensive assessment revealed 662 proteins in total in the influx blood and 809 in the efflux blood. To select candidate kidney-derived proteins from among all identified proteins, we calculated the ratio of each protein’s expression level in the efflux sample to its expression in the influx sample by using the area value, which is the integrated value of the signal intensity of the identified peptide. Consequently, depending on the protein, the expression ratio varied among the seven individuals’ samples, and for some proteins in some samples we could not calculate the expression ratio because the protein’s area value was below the lower limit for that parameter (1 × 10^6^). Therefore, we averaged the expression ratios from all seven individuals (Fig. 1a and Additional file 1: Table S1) and focused on proteins whose efflux:influx expression ratio was > 1.5 in at least five donors and > 3 in at least two donors (Table 2). The top three proteins were the three peptides that comprised the subunits of complement C1q; we therefore selected complement C1q itself as a new candidate renal secretory protein.

**Table 2:** Candidate kidney-derived proteins and their efflux:influx expression ratios in seven individuals

Protein	Donor no.							
	1	2	3	4	5	6	7	Average
Complement C1q subcomponent subunit A	3.35	4.48	4.60	9.16	3.22	2.56	38.66	9.43
Complement C1q subcomponent subunit B	6.35	12.27	0.58	21.21	5.72	2.75	14.47	9.05
Complement C1q subcomponent subunit C	7.78	10.61	4.28	17.80	6.12	2.80	7.32	8.10
Apolipoprotein(a)	2.23	13.08	1.08	2.79	4.83	2.99	16.42	6.20
Protein Z-dependent protease inhibitor	0.05	1.83	1.79	2.00	9.45	1.41	22.67	5.60
Corticosteroid-binding globulin	2.30	2.27	2.28	3.38	4.32	2.14	15.1	4.54
Lysosome-associated membrane glycoprotein 1	2.46	4.67	13.46	1.81	1.99	4.13	2.62	4.45
Phosphatidylinositol-glycan-specific phospholipase D	2.56	7.67	7.88	6.24	1.28	1.36	2.63	4.23
Plasma serine protease inhibitor	1.91	6.60	7.36	5.81	2.14	3.46	000	3.90
Apolipoprotein C-IV	4.69	8.70	2.15	4.60	0.77	1.47	3.02	3.63

The efflux:influx ratio of each protein identified by LC–MS/MS was calculated for each individual

The top 10 candidate proteins, whose efflux:influx ratio exceeded 1.5 in at least five individuals and exceeded 3.0 in at least two individuals, are indicated

We then used ELISA to measure the amount of C1q in the renal influx and efflux blood samples. C1q was increased in the efflux samples from all individuals except no. 1 (Fig. 1b). In addition, the mean serum C1q concentration was significantly higher in efflux blood (0.109 ± 0.0211 mg/mL) than in influx blood (0.0473 ± 0.072 mg/mL) (*P* = 0.0172, data not shown). These results indicate that C1q is an appropriate candidate kidney-derived protein.

### Relationship between C1q concentration and renal function in mouse models of CKD

**Fig. 2:**
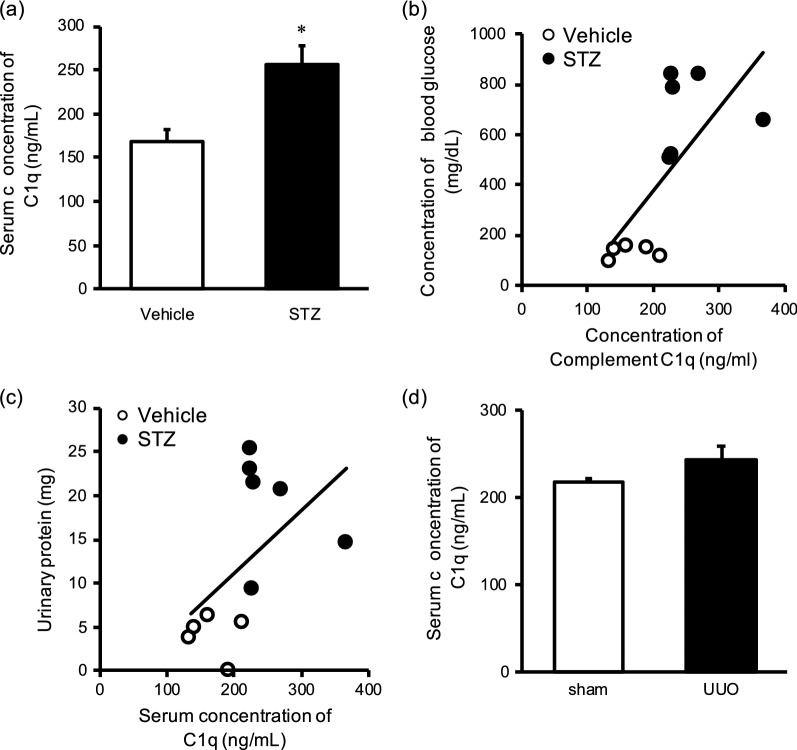
Usefulness of C1q as a biomarker of chronic kidney disease (CKD) in mice. **a** Serum concentrations of C1q in streptozocin (STZ)-induced diabetic and vehicle control mice were examined by ELISA; the mean value was significantly higher in the diabetic mice. In each mouse, the serum C1q concentration was correlated with the **b** blood glucose level and **c** amount of urinary protein. **d** Serum concentrations of C1q in unilateral ureteral obstruction (UUO) and sham-operated control mice were examined by ELISA; the mean value did not differ between groups. Data are expressed as means ± S.E.M. (n = 4–6). *, *P* < 0.05 (*t-*test) compared with value for controls

To assess whether C1q is a potential CKD biomarker that reflects renal function, we evaluated two mouse models of CKD as representatives of the wide variety of primary diseases that lead to CKD. We first looked at STZ-induced diabetic mice [12], which demonstrate diabetic nephropathy, a leading cause of CKD [13, 14]. The serum C1q concentration was significantly greater in the STZ group than in the vehicle-only control group (Fig. 2a). In addition, blood glucose concentration (Fig. 2b) and urinary protein content (Fig. 2c) both were positively correlated with serum C1q concentration (*r* = 0.679 and *r* = 0.511, respectively).

As the second model, we used UUO mice, in which renal fibrosis was induced by ligating the left ureter. Renal fibrosis is a common feature of CKD regardless of the causative disease [15], and the degree of fibrosis is correlated with disease progression [16, 17]. However, the serum concentration of C1q did not differ significantly between UUO mice and the sham-operated group (Fig. 2d). These results suggest that C1q is a biomarker that at least reflects the renal dysfunction associated with diabetes.

### Association of C1q concentration with renal function in human patients

**Fig. 3:**
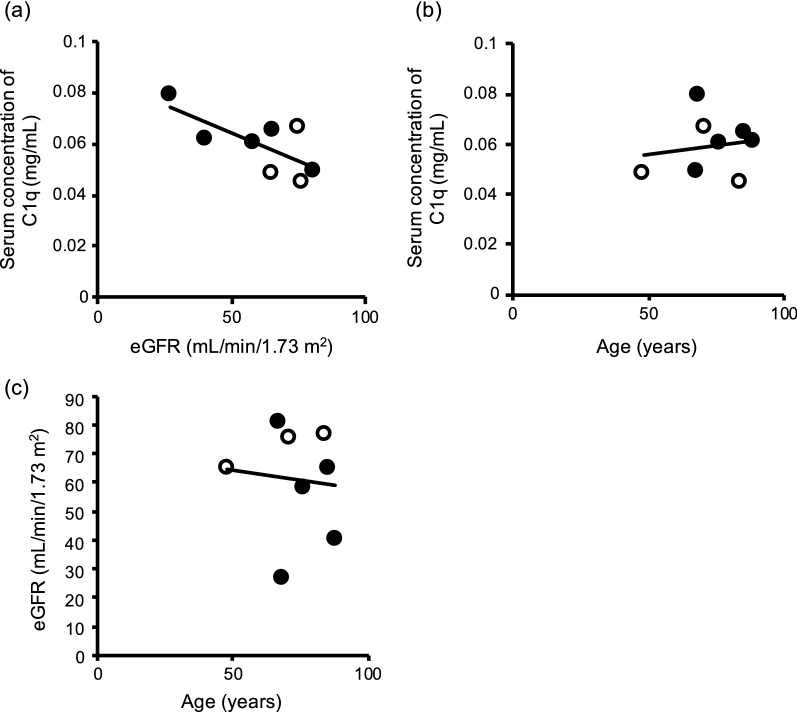
Association of C1q concentration with renal function in human patients with CKD. **a** Peripheral serum concentrations of C1q in non-CKD patients (white circles) and in patients with CKD (black circles) were examined by ELISA. The correlation between the serum C1q and estimated glomerular filtration rate (eGFR) is shown. Peripheral serum C1q concentration was not correlated with a patient’s **b** age or **c** eGFR

To evaluate whether C1q reflects renal dysfunction in humans, we measured the C1q concentration in peripheral blood serum from three non-CKD patients and five patients with CKD. When we used these samples without adjustment for donor age, serum C1q concentration tended to show a positive correlation (*r* = − 0.713) with estimated glomerular filtration rate (eGFR) (Fig. 3a). However, the blood concentration of C1q reportedly increases with age, and eGFR decreases with age [18, 19]. We therefore assessed the correlation between a patients’ age and C1q concentration (Fig. 3b) or eGFR (Fig. 3c); these analyses yielded correlation coefficients of 0.154 and − 0.100, respectively, indicating no correlation between these parameters. Overall, these results suggest that the serum concentration of C1q in humans reflects their renal function and that C1q has potential as a biomarker of CKD.

## Discussion

This research indicates that our approach of focusing on kidney-derived proteins, which are considered to sensitively reflect the state of the kidneys, is useful for identifying new CKD biomarkers; through this approach, we identified C1q is as a candidate kidney-derived protein. Although several reports have discussed the relationship between C1q and renal status, few previously discussed C1q in terms of it being a kidney-derived protein. In addition, we demonstrated that the serum C1q concentration tended to show a positive correlation with the estimated GFR of human patients, suggesting that, in humans, C1q has potential to reflect their renal function. Therefore, we hope that the approach that we developed in the current study will make it easier to identify other kidney-derived proteins with potential as CKD biomarkers.

The efflux:influx expression ratio differed between the proteome analysis and the quantitative analysis by ELISA. We consider that the differences between the quantitativeness and identification target of each method underlie the apparently inconsistent results. In the quantitative analysis by ELISA, the expression level of a protein is analyzed by using two types of antibodies that recognize different epitopes. Whereas an antibody-based identification method focuses on a specific region of a three-dimensional protein, a mass spectrometer focuses on a specific linear peptide sequence. This methodologic difference leads to a difference in identification sensitivity and likely is responsible for the different efflux:influx expression ratios that we obtained through proteomics analysis compared with ELISA.

To develop C1q as a biomarker of the chronic inflammatory pathology of CKD, the constitutive role of C1q in the kidney and the mechanism of its increased expression in renal dysfunction must be understood. C1q is the first component of the classical pathway of the complement system [19] and is primarily produced by activated dendritic cells [20]. Therefore, regarding the renal secretory mechanism of C1q, the increased C1q concentration associated with CKD may reflect the infiltration of macrophages into the kidney.

In addition, the sensitivity and specificity of a candidate biomarker for a target disease are important. From the viewpoint of sensitivity, blood levels of C1q can be quantified, demonstrating its suitability for minimally invasive blood tests. However, from the viewpoint of specificity, blood levels of C1q are increased in rheumatoid arthritis [21] and sarcopenia [22]. Furthermore, considering that CKD is a consequence of numerous and diverse primary diseases with complicated underlying pathology, it will be difficult to diagnose CKD solely on the basis of blood levels of C1q. Therefore, accurate diagnosis of CKD will require the use of multiple biomarkers, including C1q [23, 24]. The approach we developed in the current study likely will be useful for identifying other kidney-derived proteins with potential as CKD biomarkers.

Regarding the relationship between C1q and the kidney, the clinical condition of C1q nephropathy is associated with profound protein loss through the urine and the deposition of C1q in the kidney [25, 26]. The mechanisms underlying the onset of C1q nephropathy and renal deposition of C1q have not yet been clarified, but the C1q deposits reportedly activate the renal immune system, leading to an inflammatory immune response and inflammation-induced renal damage [27, 28]. Given that the induction of an inflammatory response is involved in the progression of CKD and that, compared with healthy subjects, CKD patients have increased blood cytokine levels [29, 30], perhaps C1q acts as an exacerbating factor in the inflammation-associated decline of renal function during CKD. We plan to use various mouse models of CKD to disclose the role of C1q in the kidney.

Moreover, CKD—like arteriosclerosis, decreased skeletal muscle mass, and cognitive dysfunction—can be regarded as a marker of aging at the individual level [31]. In this regard, the klotho/Wnt/β-catenin pathway is known to be involved in the renal fibrosis of aged kidneys and of CKD, because klotho expression is reduced in aged or impaired kidneys [32] and because the activation of Wnt signaling is responsible for the aging phenotype in klotho mice [33]. Given that C1q is involved in the activation of Wnt signaling activation in vivo [19], C1q-induced activation of Wnt signaling conceivably may be involved in aging and aging-related organ or tissue dysfunction. In addition, the majority of patients with CKD die from cardiovascular disease (CVD) before progressing to end-stage renal disease [34, 35]. CKD-associated CVD typically leads to clinical outcomes such as sudden cardiac death and hospitalization for heart failure [36]. Considerable effort has been devoted to investigating the etiology and pathogenesis involved in the progression of CKD-associated CVD, including the retention of uremic toxins and inflammation [37], but the high-risk factors and their underlying mechanisms are still far from being elucidated. However, given that C1q may promote aging-associated arteriosclerosis [38], the increased levels of C1q in patients with CKD might contribute to the etiology of CKD and its pathogenesis, including the progression of CVD. Together, these previous findings and our current results indicate the potential usefulness of C1q as a biomarker for the chronic inflammatory pathology of CKD.

## Conclusion

Our results indicate that our approach of focusing on kidney-derived proteins is useful for identifying new CKD biomarkers and that C1q has potential as a biomarker of renal function.

## Supplementary Information

**Additional file 1:** **Table** **S1.** Identified kidney-derived proteins and their efflux:influx expression ratios in seven individuals.

## Data Availability

All data generated or analyzed during this study are included in this published article.

## Abbreviations

CKD: Chronic kidney disease
CVD: Cardiovascular disease
eGFR: Estimated glomerular filtration rate
STZ: Streptozocin
UUO: Unilateral ureteral obstruction
