# Supplementary material for: Identification of biomarkers of chronic kidney disease among kidney-derived proteins
Source: Clin Proteomics. 2022 Jan 11;19:3. doi: 10.1186/s12014-021-09340-y (PMC8903635; doi:10.1186/s12014-021-09340-y)
Supplement: Supplementary file 1 — Additional file 1: Table S1. Identified kidney-derived proteins and their efflux:influx expression ratios in seven individuals. [file 12014_2021_9340_MOESM1_ESM.doc]

**Supplementary Table 1. Identified kidney-derived proteins and their efflux:influx expression ratios in seven individuals.**

Donor no.

Protein 1 2 3 4 5 6 7 Average

Contactin-1 0.00 3.33 4.03 198.89 1.37 148.02 0.96 50.94

Dynein heavy chain 5, axonemal 2.44 15.34 0.00 1.14 0.02 0.88 52.35 10.31

Complement C1q subcomponent subunit A 3.35 4.48 4.60 9.16 3.22 2.56 38.66 9.43

Complement C1q subcomponent subunit B 6.35 12.27 0.58 21.21 5.72 2.75 14.47 9.05

Complement C1q subcomponent subunit C 7.78 10.61 4.28 17.80 6.12 2.80 7.32 8.10

Metalloproteinase inhibitor 1 0.00 2.00 0.00 0.00 0.86 49.61 0.00 7.50

Complement C1r subcomponent-like protein 0.69 1.58 0.62 18.14 3.98 1.41 20.55 6.71

Apolipoprotein(a) 2.23 13.08 1.08 2.79 4.83 2.99 16.42 6.20

Protein Z-dependent protease inhibitor 0.05 1.83 1.79 2.00 9.45 1.41 22.67 5.60

Rho GDP-dissociation inhibitor 2 0.00 0.00 0.00 0.00 35.78 1.48 0.00 5.34

The efflux:influx ratio of each protein identified by LC-MS/MS was calculated for each individual. The top 10 identifeid proteins are indicated.
